# Formidable challenges to the notion of biologically important roles for dietary small RNAs in ingesting mammals

**DOI:** 10.1186/s12263-017-0561-7

**Published:** 2017-07-07

**Authors:** Stephen Y. Chan, Jonathan W. Snow

**Affiliations:** 1 Department of Biology, Barnard College, New York, NY 10027 USA; 2 Center for Pulmonary Vascular Biology and Medicine, Pittsburgh Heart, Lung, Blood, and Vascular Medicine Institute, Department of Medicine, University of Pittsburgh School of Medicine and University of Pittsburgh Medical Center, BST 1704.2, 200 Lothrop Street, Pittsburgh, PA 15261 USA

**Keywords:** sRNA, Cross-kingdom, Ecology, Genetically engineered, Biotechnology, Agriculture, Diet, Invertebrate, miRNA, Plant

## Abstract

The notion of uptake of active diet-derived small RNAs (sRNAs) in recipient organisms could have significant implications for our understanding of oral therapeutics and nutrition, for the safe use of RNA interference (RNAi) in agricultural biotechnology, and for ecological relationships. Yet, the transfer and subsequent regulation of gene activity by diet-derived sRNAs in ingesting mammals are still heavily debated. Here, we synthesize current information based on multiple independent studies of mammals, invertebrates, and plants. Rigorous assessment of these data emphasize that uptake of active dietary sRNAs is neither a robust nor a prevalent mechanism to maintain steady-state levels in higher organisms. While disagreement still continues regarding whether such transfer may occur in specialized contexts, concerns about technical difficulties and a lack of consensus on appropriate methods have led to questions regarding the reproducibility and biologic significance of some seemingly positive results. For any continuing investigations, concerted efforts should be made to establish a strong mechanistic basis for potential effects of dietary sRNAs and to agree on methodological guidelines for realizing such proof. Such processes would ensure proper interpretation of studies aiming to prove dietary sRNA activity in mammals and inform potential for application in therapeutics and agriculture.

## Background

There is ongoing debate about the putative transfer and ensuing regulation of gene activity by diet-derived small RNAs (sRNAs) in ingesting mammals. Proof of active and reproducible uptake of diet-derived sRNA could profoundly guide our understanding of oral therapeutics and nutrition, the safe use of RNA interference (RNAi) in crop biotechnology, and ecological relationships of organisms. In this review, we make the case that insufficient evidence currently exists to support a biologically relevant impact of sRNAs in dietary material on gene expression of ingesting organisms, specifically mammals. sRNAs are RNA molecules of <200 nucleotides in length that are typically involved in regulating other cellular processes. sRNAs include microRNAs (miRNAs), short-interfering RNA (siRNAs), and longer double-stranded RNAs (dsRNAs) from which siRNAs can be derived. Specifically, siRNAs and miRNAs are involved in the post-transcriptional regulation of gene expression in animals through a process known as RNA interference (RNAi) (reviewed in [1]). While these two RNA biotypes are processed and act similarly via RNAi-mediated mechanisms throughout the plant and animal kingdoms, their origin is distinct. miRNAs are encoded by endogenous genes, while siRNAs are usually generated from double-stranded RNAs (dsRNAs) that are introduced to the cell from an exogenous source or from less well-characterized endogenous sources. After processing, both miRNAs and siRNAs bind specific complimentary sequences in messenger RNA transcripts and regulate gene expression through the repression of translation and/or degradation of the targeted mRNA (reviewed in [2]).

Uptake of diet-derived sRNAs with resulting actions on gene expression of an ingesting organism was first described in *Caenorhabditis elegans* [3, 4]. Here, when dsRNAs were added to the diet or expressed in bacteria that make up the diet of this organism, these RNAs were found to silence multiple genes after serving as the template for siRNA formation. Since those reports, studies of oral exposure of various invertebrate organisms to dietary material containing in vitro synthesized dsRNAs or artificially expressing dsRNAs have demonstrated that various invertebrate organisms take up sRNAs from diverse dietary sources. Notably, failure of many invertebrate species to take up dietary sRNA efficiently has been described in both artificial [5–7] and natural ([8–13] and reviewed in [14]) contexts, underscoring the species-dependent variability in this process.

Historically, multiple studies confirmed that artificial sRNAs, such as siRNAs, had little capacity to translocate through the mammalian gut when naked and unmodified [15]. When considering the variable uptake of dietary sRNAs in invertebrates as well as the more complex anatomic barriers in the mammalian gut, it was thought that transfer of naturally occurring sRNAs from dietary material to ingesting mammals would be minimal. Therefore, when transfer of diet-derived small RNAs in ingesting organisms in a natural context was first reported by C-Y Zhang and colleagues [16], it generated substantial interest. Concurrently, the presence of sRNAs from exogenous sources was detected in human plasma [17], and the hypothesis of transfer of sRNAs between organisms gained significant attention [18–20]. While these studies suggested the possibility of cross-kingdom communication mediated through the diet [21–24], other subsequent studies provided considerable evidence that systemic uptake of ingested miRNAs from a different species is negligible in mammals [9, 10, 25, 26] and below levels required to be biologically relevant when acting through canonical sequence-specific miRNA-mediated mechanisms. Controversy remains, with a number of groups offering data and interpretations in support or in opposition of this phenomenon [27–68].

Biologic and technical reasons may both be at play in leading to differing results and interpretations [38, 48]. Ongoing disagreement primarily centers on the prevalence, magnitude, and, most importantly, the activity of sRNAs from dietary sources. In this review, we integrate information garnered from studies using dietary delivery of sRNA in mammals as well as studies of sRNA function in mammals, invertebrates, and plants. We offer our viewpoint of what is known in this controversial field. We also highlight the challenges of demonstrating uptake and activity of sRNAs in recipient mammals, particularly in light of substantial biologic obstacles that likely inhibit transfer of intact dietary sRNAs and our collective lack of mechanistic insight into how sRNAs might overcome these obstacles during normal ingestion. Furthermore, concerns about technical challenges and the absence of consensus on appropriate methods have led to reservations regarding the robustness, reproducibility, and biologic significance of some findings. In light of those issues, for any continuing investigation to impact this field, concerted efforts should be made to develop a strong mechanistic model as well as a consensus for methodologic guidelines for ultimate proof or dismissal for this controversial hypothesis.

## Implications

The biologic activity of diet-derived sRNAs in ingesting vertebrate species could have significant implications for a number of fields. First, there is substantial interest in using circulating sRNAs derived from the diet as biomarkers [69] and the potential to harness uptake of diet-derived sRNA by mammals could represent a powerful new therapeutic strategy for the treatment of disease [70]. The ability to enhance existing systems for natural uptake of diet-derived sRNA by mammals would provide an attractive starting point for such endeavors. Yet, even if natural uptake only occurs at levels too low to be biologically meaningful, it is likely that some obstacle to therapeutic uptake could be overcome, as the following examples illustrate. Some evidence suggests that artificial “exosome” lipoplexes [71] or plant nanoparticles [72] can protect sRNA from degradation in the digestive tract [73]. Modifying nanoparticles with antibodies to specific surface proteins on recipient cells can enhance targeting and uptake of sRNA [74]. In addition, passage across the digestive tract barrier might be increased through the use of pharmacologic enhancers of intestinal permeability [75] or engineered bacteria [76]. Recently, it was shown that plant nanoparticles [77] and modified lipophilic siRNA molecules [78] can be engineered to allow “homing” to distal sites and siRNA-mediated activation of immune pattern recognition receptors can be inhibited by 2’ modification of nucleic acid moieties [79]. Second, agriculture could potentially be transformed in the coming years by RNAi-based technologies which take advantage of cross-kingdom sRNA transfer, including genetically engineered (GE) plants and topical sprays [80, 81]. However, if systems indeed exist in mammals for natural uptake of diet-derived sRNAs, it would alter assumptions upon which these new technologies have been built and tested [82, 83]; the most important being the minimal risk to mammals due to negligible uptake and transfer [84, 85]. Third, the existence of robust cross-kingdom regulation of gene expression via ingested sRNAs could carry substantial ecologic significance. In fact, a fundamental implication of this hypothesis is that some type of co-evolution has driven this relationship between diet and ingesting organisms. Cross-kingdom interactions might be expected to impact predator-prey interactions [86] or zoopharmacognosy, defined as self-medication by animals [87], with implications for ethnobotany and the use of traditional medicine in human societies [88]. Thus, natural uptake of diet-derived sRNA by mammals could expose an exciting new layer of communication in these relationships.

## Current state of the field

While the potential impacts described above are exciting, definitive proof that dietary sRNAs are routinely taken up by the ingesting mammal, are transported, and have biologic action on gene expression is wanting. The initial report by C-Y Zhang and colleagues [16] reporting that miRNAs from rice were taken up by ingesting mice with subsequent modulation of gene expression prompted a number of key questions [22, 23]. First, could the existence of exogenous, diet-derived, sRNAs in mammalian tissues be a common phenomenon and were the amounts observed biologically relevant? Second, what systems must exist for efficient uptake and function of exogenous, diet-derived, sRNAs?

Two studies immediately addressed the prevalence of diet-derived sRNAs by using RNA-sequence datasets from diverse mammalian organisms. While both found sequences corresponding to plant miRNAs in these datasets, the conclusions were dramatically different. One group suggested that the results provided considerable support for the natural uptake of sRNAs from the diet [17], while the other suggested that observation of diet-derived sRNAs was due to artifact [9]. Similarly, groups attempting to confirm the findings of Zhang et al [16] through feeding experiments did detect diet-derived sRNAs, but concluded that the levels in the tissue and even in the diet were well below the levels required to be biologically relevant [10, 25, 26]. While the studies above have largely focused on miRNAs from a different species (xenomiRs), it is worth noting that a number of groups have also been interested in whether sRNAs found in milk [89] might be passed on to the offspring through the diet.

Subsequent studies, using dataset analysis of animal tissues and fluids or feeding experiments, have largely agreed that sRNAs from dietary sources (both within and between species) can be observed in mammalian tissues and dietary material. However, contributing groups have fallen into two distinct camps when drawing conclusions regarding whether the level detected can be construed as biologically significant [9, 17, 27, 29, 30, 33, 34, 43, 45, 46, 51, 54–56, 58, 59, 66, 68, 90] or artifactual [9, 10, 25, 26, 28, 37, 39, 41, 42, 44, 49, 50, 65, 67].

Recent studies supporting biologically relevant uptake have focused on the plant-derived small sRNA MIR2911 [54–56, 59, 90]. Two of these studies also measured levels of MIR2911 in body fluids and found 1189 fM in plasma [90] or 207 fM in serum [59] after feeding. Assuming 1.46 ml of total blood, 52.2% of which is plasma (and slightly less is serum), these levels are equal to 3.7 × 10^8^ or 6.4 × 10^7^ per mouse or 0.0026 or 0.00045 copies per cell in this mouse. These values, which are in line with our own findings and those of others groups [10, 25, 26], suggest that unless some unknown mechanisms are involved, insufficient levels are present to be pervasively active by canonical mechanisms. It is important to note that the circulatory levels of miRNAs may not be the most precise gauge of whole-body miRNA content, especially given the possibility of localized enrichment of miRNAs in specific tissues or cell types. In the most recent study in this field, Kang and colleagues came to similar conclusions after combining exhaustive dataset analysis with carefully controlled feeding experiments [65]. Examination of sRNAs in >800 datasets from human tissues and body fluids revealed that although dietary sRNAs were commonly detected, they were present at levels of ~5 copies per cell [65], far below the levels shown for their endogenous counterparts, which may reach 50,000 copies per cell for some miRNA entities [91]. Feeding experiments using different plant diets in rats and different milk diets in pigs did not find any evidence of substantial uptake of dietary sRNA. This newest report represents the most rigorous assessment of diet-derived miRNAs to date. Accompanied by prior data from independent groups [10, 25, 26], this collective body of work concludes that uptake and canonical activity of dietary miRNAs are neither a prevalent nor robust mechanism in mammals. However, whether such transfer may occur in specialized contexts is still debated. To prove that point, a much more solid mechanistic framework and consensus on methodologic guidelines for proof are essential.

## Defining a more solid mechanistic gramework for investigation

**Fig. 1:**
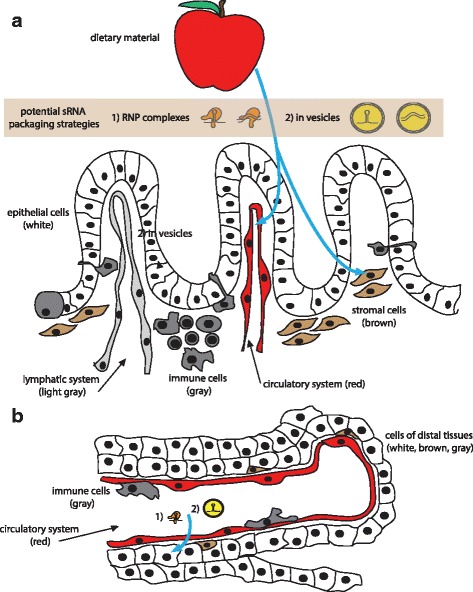
Model for uptake of dietary sRNA from the digestive tract. To carry RNAi regulatory activity on gene expression in an ingesting organism, **a** sRNAs from the diet (potentially packaged in (*1*) ribonucleoprotein (RNP) complexes or (*2*) in vesicles) should cross the epithelial cell (*white*) barrier via transcellular or paracellular mechanisms or via conveyance by immune cells (*gray*). They should then be taken up by proximal cells, such as stromal cells (*brown*) or must gain access to the circulatory (*red*) or lymphatic system (*light gray*) for systemic dissemination. **b** Subsequently, after exit from the circulatory system (*red*), uptake of sRNAs would ensue by cells of various tissues and organs (*gray*, *brown*, and *yellow*). None of these putative steps are understood at the level of molecular mechanism

**Fig. 2:**
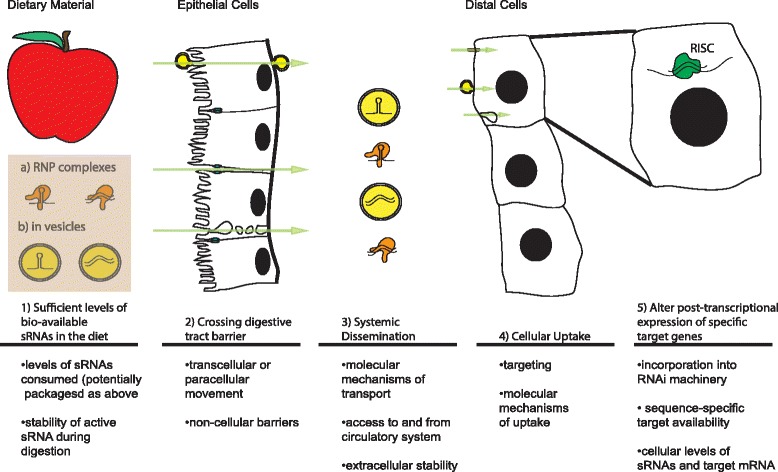
Critical steps for successful alteration gene expression of an ingesting organism by dietary sRNA (adapted from [64]). (*1*) Sufficient levels of bio-available sRNA in the diet (potentially packaged (*a*) in ribonucleoprotein (RNP) complexes or (*b*) in vesicles); (*2*) crossing the digestive tract barrier; (*3*) systemic dissemination; (*4*) cellular uptake; and (*5*) alteration of post-transcriptional expression of specific target genes by the RNA-induced silencing complex (RISC, *green*)

Significant biologic hurdles exist for dietary sRNAs to engage recipient mRNA transcripts and affect gene expression directly in ingesting organisms. Furthermore, we have a nearly complete absence of mechanistic insight into how these barriers could be overcome. A number of discrete steps must be considered and explained if a given sRNA in the diet indeed has the potential to alter the gene expression in an ingesting mammal (Figs. 1 and 2).

We can address these considerations separately as five questions.

1. Are there sufficient levels of bio-available sRNAs in the diet?
2. Do sRNAs cross the digestive tract barrier?
3. Are sRNAs disseminated systemically?
4. Is there cellular uptake of sRNAs?
5. Can delivered sRNAs alter the post-transcriptional expression of specific target genes?

### Are there sufficient levels of bio-available sRNAs in the diet?

Theoretically, three conditions must be met for sufficient bio-available sRNAs to be consumed in the diet by an ingesting organism to impact gene expression. First, an animal must be likely, and physically able, to consume enough of the diet to ingest a biologically relevant amount of a given sRNA. Second, this sRNA must be able to endure the harsh environment of the digestive tract. Third, a surviving sRNA must retain biologic activity.

The first condition, whether an animal can consume sufficient amounts of a given sRNA in a normal diet, is affected by a number of variables. The amounts of specific sRNA types found in different dietary material are quite divergent, as exemplified by wide variation of plant miRNA levels in a relatively small set of plant species examined [53]. For example, plant MIR156a exists at 5 × 10 [6] copies per milligram of cantaloupe tissue, but 1000 copies per milligram of apple tissue [10]. In addition, sRNA expression is known to vary significantly even among different plant tissues in a given species and are highly sensitive to environmental conditions [92], such as in fruit during ripening [93, 94]. Plants also possess a number of other sRNAs, which are generated from longer dsRNA precursors, including hairpin-derived siRNAs, natural antisense siRNAs, secondary siRNAs, and heterochromatic siRNAs [92, 95]. In addition, rRNA or tRNAs and their degradative fragments can exist in at high levels and it is possible that these RNA species may have biological activity [64]. In the few species for which these other sRNA types have been extensively examined, the levels also appear to be present over a wide range. Perhaps a more illuminating fact highlighting the challenge of ingesting the appropriate amount of dietary RNAs for canonical activity is the limiting levels of dietary sRNAs consumed by any animal [96]. For example, recent estimates of fruit and vegetable intake in Europeans range from 103 to 454 g per day [97]. Based on the 6 × 10^6^ copies of MIR156a found per milligram of cantaloupe, a person would need to consume an untenable 1670 kg of cantaloupe in order to reach the minimum of 100 copies per cell (assuming 100% uptake and uniform transfer to cells) [10]. The amount of sRNA required to achieve biologically relevant effects on gene expression is currently thought to be 100–10,000 copies per target cell, dependent on the amount of target transcript [98–100]. While a number of other factors, such as the rate of consumption and the half-life of ingested sRNA, are likely to impact actual per cell amounts, measurement of sRNA in tissues supports the contention that dietary amounts are typically too low to be relevant. However, cases do exist where the diet may theoretically contain enough sRNAs to be biologically meaningful. For example, MIR2911 species was found at 5000 fm/g in honey suckle [56, 90] and ~228 fm/g in spinach [59], corresponding to 3 × 10^12^ and 1.3 × 10^11^ copies per gram, respectively. Using a calculated estimate of the number of cells in a 25 g mouse of 1.42 × 10^11^ cells (adapted from [101] where a 70 kg human = 40 × 10^13^ cells), this mouse, with a typical diet of 4–5 g per day, could theoretically consume the 4.7 g of honeysuckle, but not the 109 g of spinach required to provide 100 copies per cell assuming 100% transfer. Yet, MIR2911 appears atypical in its high amounts in plant tissue and stability characteristics relative to other MIRNA.

Thus, current data suggest that in the vast majority of cases, dietary material does not contain enough sRNA to feasibly enable uptake of biologically meaningful levels. This view could be changed if cellular systems for specific transport, amplification, or concentration existed. While some invertebrate species possess a system that amplifies a primary siRNA to more numerous progeny siRNAs [102], no evidence for such an amplification system has been found in mammals [18].

Second, ingested sRNAs must be able to withstand harsh extracellular environments, particularly the mammalian digestive tract, where oral bio-availability of intact macromolecules is typically very low. Various types of processing of dietary material, such as cooking, could also impact the survival and activity of sRNAs in food. Such processing has been found to result in RNA breakdown in some instances, but not in others. For example, miRNAs found in olives [103] are not detected in olive oil [52]. In addition, although levels of miRNAs contained in milk are relatively stable [104], they are reduced by processing and storage [49]. Such principles vary based on context, as dietary miRNAs from bovine meat appears less sensitive to multiple processing methods [51]. Perhaps more importantly, independent of food processing before ingestion, the highly proteolytic context of the mammalian digestive tract itself allows only 1–2% of proteins, whether packaged in lipids or not, to remain intact and bio-available after digestion [105]. With regards to sRNA, pancreatic ribonucleases, which are a major enzyme in the digestive tract [106], are very efficient at degrading dsRNA [107]. Interestingly, there is some evidence that this enzyme exists to degrade dsRNA for defense against the biological effects of these molecules [108]. Yet, our understanding of the rate and mechanisms of sRNA degradation is incomplete, both intracellularly [109] and extracellularly. Some studies have begun to directly assay sRNA stability in simulated digestive environments [110, 111] or in vivo [56] in mammals. Thus far, these reports suggest great complexity in determinants of sRNA degradation, based on both digestive tract tissue architecture and specific environments that have evolved in mammals in a diet-dependent manner [112].

Any resistance to degradation of dietary sRNAs in the extracellular space relies greatly on specialized packaging and modifications. In theory, packaging of sRNAs could occur in a manner that has been selected for cross-kingdom communication. If so, this should have arisen due to evolutionary pressure based on an ecological relationship between the ingested and ingesting organisms. While the understanding of extracellular transport of sRNAs in plants is still incomplete (reviewed in [113]), preliminary evidence suggests that packaging strategies appear similar to those described in mammals, where extracellular sRNA is transported after incorporation in exosomes/microvesicles or inclusion in ribonucleoprotein complexes (reviewed in [64]). Exosomes or microvesicles are a heterogeneous group of membrane-bound vesicles that can be released from the cell as part of a regulated process to allow delivery of diverse macromolecules to other cells within an organism [114]. Plants may possess exosome-like particles, known as nanoparticles [72], which can contain sRNAs, lipids, and proteins. First described in grapefruit [115], these exosome-like particles have been theorized to provide a mechanism for communication between plants and animals [116]. However, while these can carry sRNAs [72], as of yet, they have not been shown to deliver bioactive sRNA to cells. In addition, since these nanoparticles are produced artificially during destructive mechanical processing of plant material and then concentrated, it is unclear whether they are present in the native plant or whether they could be naturally released in amounts that would protect and deliver meaningful levels of sRNAs assuming 100% uptake and uniform transfer to cells. In addition to nanoparticles, sRNAs complexed with proteins have been found in the vascular systems of plants [117] and animals [64] and appear to provide stability to sRNAs in an extracellular environment. Additional mechanisms, such as the covalent modifications found on many sRNA molecules [92, 118], may also protect sRNAs. There is some evidence that stability differs among sRNA species. For example, MIR2911 appears unique among sRNAs examined in its ability to withstand degradation in vitro and within the mouse digestive tract [59]. This sRNA species is extra-exosomal, associated with a ribonucleoprotein complex, and rich in GC sequences. Some or all of these principles may contribute to stability [59].

Third, any ingested sRNA must retain biologic activity. Yet, contemporary studies have relied on quantitations of total amounts of ingested sRNAs without any measurement of remaining activity (i.e.*,* direct binding to target mRNAs with consequent effects on translation or mRNA degradation). As such, this may have led to erroneous conclusions about the impact of a given process on the subsequent biologic potential of any given dietary sRNA.

### Do sRNAs cross the digestive tract barrier?

The highly selective barrier of healthy gut epithelial tissue [119], which in mammals is comprised of mucus in addition to the epithelial cells themselves, provides a severe impediment to uptake of environmental sRNA (Fig. 1a). Our current understanding defines two possible modes of transport across the digestive tract epithelium, either transcellular or paracellular [119]. Epithelial cells themselves regulate transcellular permeability via transport pathways through their cytoplasm, including transcytosis and via protein transporters. Microvesicles or exosomes could also fuse with the epithelial cell membrane. On the other hand, paracellular permeability requires transport between the epithelial cells and is strictly regulated by tight junctions under normal circumstances, rendering this path unlikely.

The majority of our information about the mechanism for dietary uptake of sRNA is derived from invertebrates. *C. elegans* utilizes a system involving the SID-1 dsRNA channel as well as a number of additional proteins involved in endocytosis, including the gut-specific SID-2 and the SID-5 endosomal factor (reviewed in [120]). Other data in invertebrates suggest that endocytosis may be a common mechanism for sRNA uptake by the cells of the digestive tract (reviewed in [14]). Passage of molecules across the gut epithelium is also mediated by transcellular and paracellular transport in mammals. However, there are limited data in support of either mechanism for sRNA uptake. Some studies have suggested that milk exosomes are endocytosed by mammalian cell lines, perhaps as the first stage for transcytosis [121–123], but there are currently no compelling data in support of paracellullar transport of sRNAs under normal conditions. There is potentially illuminating research in the field of microbiology, where the transfer of sRNA between an infectious agent and host may be common [20]. A recent report demonstrated that exosomes containing sRNA released by the gastrointestinal nematode *Heligmosomoides polygyrus* were targeted to intestinal cells in mice [124]. In addition to intestinal epithelial cells [119], the mammalian digestive tract is colonized by a variety of immune cells, including M cells, B cells, T cells, macrophages, and dendritic cells [125], and these could play a role in the uptake of sRNA from dietary sources. Plant nanoparticles described above [115] can target intestinal macrophages [116, 126]. While these particles do seem to have effects on local tissue, these mechanisms do not appear to be sRNA-dependent, and no evidence to date has demonstrated immune delivery of sRNA in such nanoparticles to other recipient cells in vivo.

sRNA uptake in mammals could be influenced by both normal and pathogenic changes in the barrier properties of the digestive tract. For example, barrier function in humans has been shown to decrease with age likely due to increased paracellular permeability [127]. Pathogenic changes in barrier function, such as those caused by xenotoxicity [128], inflammation [129], or infection [130], could also change the efficiency of sRNA passage across the digestive tract. For example, the Cholera toxin released by *Vibrio cholerae* during infection results in cell junction dysfunction and a significant increase in paracellular permeability [131]. Correspondingly, dietary uptake of some sRNA that occurs in healthy individuals [56] has been reported to be enhanced by intestinal injury, possibly via increased paracellular permeability [55]. While all of these scenarios are possible, there has been no conclusive proof that putative uptake is biologically relevant or happening at high enough levels under any circumstances. Taken together, no mechanisms for the transfer of sRNA across the epithelium of the digestive tract of vertebrates have been described at the molecular level. Such understanding is essential to advance the field beyond descriptive phenomenology. First, uptake of diet-derived sRNA in the epithelial tissues and cells of ingesting organisms should be pursued using methods that do not require amplification for detection, such as labeled molecules [132], in situ hybridization, and engineered cellular detectors (reviewed in [133]). Second, through rigorous genetic and pharmacologic gain- and loss-of-function experiments, it is critical to determine if any sRNA transport system exists that could mediate proposed transport across this formidable barrier.

### Are sRNAs disseminated systemically?

Once across the barrier of the digestive tract epithelia, sRNAs must be able to survive the internal environment and either be taken up by cells that are proximal to the digestive tract (Fig. 1a) or be spread systemically (Fig. 1b). In the context of mammals, systemic spread would be very complex and require multiple rounds of uptake and dispersal by intermediate cells or crossing of cellular barriers to reach the distal tissues (Fig. 1b).

In vertebrates, extracellular sRNAs have been extensively characterized. Specifically, miRNAs can be secreted to regulate gene expression in a non-cell-autonomous manner and are relatively stable due in part to special processing [134]. miRNAs have been shown to be incorporated into a variety of ribonucleoprotein complexes, including those containing ARGONAUTE family members [135–137], HDL [138, 139], and HuR [140], which provide stability and potentially aid uptake in specific target cells. In addition, miRNAs can be delivered by a diverse cohort of lipid-bound vesicles, including exosomes, in a wide range of biologic processes [141–148]. Sorting of miRNAs into exosomes can be influenced by a number of factors (reviewed in [149]). For example, genetic or pharmacologic manipulation of the sphingolipid metabolism enzyme, nSMase2, can impact the efficiency of miRNA incorporation into exosomes [143]. The function of extracellular vesicles in intercellular communication is still not fully understood [133], and controversy still exists regarding the exact contribution of exosomes in the intercellular spread of sRNAs [150]. For example, one study found that there was far less than one molecule of a given miRNA per exosome [151], making it difficult to envision delivery of meaningful amounts. However, other recent studies provide more convincing evidence supporting biological relevance of exosomal miRNA delivery (e.g., [152]). Furthermore, the continued emergence of studies showing transfer of active miRNAs via exosomes from tissue to tissue in vivo supports a more significant role [133]. Even less well understood, two other modes of sRNA spread have been documented in vertebrates, including via gap junctions [153–155] and cell bridges [156].

These mechanisms of intercellular communication by endogenous sRNAs appear well suited to link cells that are relatively close together, similar to the manner of a paracrine hormone. It seems less likely that sRNAs are efficient in communicating with cells at a considerable distance, but studies in that regard are still in progress. For dietary sRNAs to function in the same way, the obstacles are considerable. To reach the distal tissue from the digestive tract, sRNA would have to travel through one of two dissemination systems, the circulatory system or the lymphatic system, both of which are encapsulated in selective cellular barriers. Thus, transport across these cellular barriers would require multiple rounds of paracellular and/or transcellular transport [157]. Furthermore, such transport would need to avoid known endogenous mechanisms that clear proteins-complexes, exosomes, and cellular debris from circulation. For example, the RNAse1, a pancreatic RNase with potent activity against dsRNA, is secreted by endothelial cells [158], likely contributing to destruction of dsRNA in circulation. Finally, how such sRNAs would target specific cells for delivery has not been described. In terms of extracellular vesicles in vivo, distribution is determined by cell source [159], suggesting that some factor in the originating cell contributes. Surface receptors involved in homing have been demonstrated in cancer-derived vesicles [160] but not in normal cells (reviewed in [133]). Work studying the transfer of sRNA from parasite to mammalian host may be able to provide some insight [161]. For example, the *Trypanosoma cruzi* parasite has been reported to shed sRNA in extracellular vesicles that can transfer these sRNA species to mammalian cells [162]. Alternatively, since the mammalian digestive tract is colonized by a variety of immune cells [125], these cells could be involved in both uptake and systemic dissemination through their migration through the lymph system. However, as immune cells from the periphery, such as the digestive tract, typically home to lymphoid organs to communicate with other immune cells, this mechanism is less likely to facilitate widespread delivery to non-immune cells.

In total, if existent, systemic spread of sRNA in mammals would have to rely on complex and repeated rounds of uptake and dispersal by intermediate cells or crossing of cellular barriers. For example, one recent study reported the existence of plant miRNAs in mammalian breast milk exosomes [66], suggesting that plant sRNAs undergoes a minimum of four rounds of transport through cellular barriers from plant diet to breast milk. Yet, another group reported that these plant miRNAs in breast milk merely represent technical artifacts and contamination [67]. Thus, without further delineation of a putative underlying mechanism for transport, currently available data do not rule out the possibility of sRNA dissemination in specific contexts yet offer no conclusive proof of such transport and are persistently questioned regarding the possibility of technical artifact.

### Is there cellular uptake of sRNAs?

Our knowledge about the mechanisms responsible for mediating sRNA uptake by cells in distal parts of the organism is also largely derived from invertebrates, with little data reported in mammals. In invertebrates, entry into cells outside of the digestive tract occurs via a dsRNA channel as in *C. elegans* SID-1 or through clathrin-mediated endocytosis as in *Drosophila melanogaster* [163, 164]. Notably, a SID-1 homolog exists in vertebrates, and it may be involved sRNA uptake in humans [165, 166]. Receptors that interact with ribonucleoprotein complexes containing sRNAs may facilitate uptake via endocytosis. For example, miRNAs complexed with HDL can be endocytosed after interaction with the receptor SRBI [138]; although, the biologic significance of this event has been questioned [139]. Cellular machinery involved in the uptake of sRNAs in extracellular vesicles is still not fully defined, with both clathrin-mediated and calveolin-dependent mechanisms being implicated [167].

Cells of different tissues within an organism may also have different potential for uptake. For example, lipid dyes used to label milk-derived exosomes are preferentially taken up by the liver and spleen after intravenous injection [123, 168]. However, escape of extracellular sRNAs from the endosome to the cytoplasm may, in fact, be the most limiting factor [169, 170]. Once endocytosed, sRNAs can be recycled back to the extracellular space, be degraded in the lysosome, or exit the endosome via incompletely understood mechanisms [171, 172]. Further studies to define the processes governing sorting for endogenous extracellular RNAs would provide a better understanding of the feasibility of the proposed handling of diet-derived sRNA. In addition, recent evidence suggests that exosome-delivered miRNA is specifically targeted for degradation by the XRN1 nuclease [173].

### Can delivered sRNAs alter the post-transcriptional expression of specific target genes?

Even if a dietary sRNA could traverse the above conditions intact, three additional points would have to be met to initiate canonical post-transcriptional regulation of specific target genes (Fig. 2). First, the RNAi machinery of the cell must recognize foreign sRNA molecules. Evidence suggests that inclusion of sRNAs in active RISC complexes is highly regulated and may be coupled to processing [1]. Although studies have shown that transfected or overexpressed xenomiRs can engage mammalian mRNA targets in cell culture, it is not clear that sRNA molecules from other species possess the requisite characteristics for recognition and efficient use by the RNAi machinery in ingesting organisms naturally. In addition, mammalian cells possess a number of pattern-recognition receptors that recognizes dsRNAs associated with viral infection [174]. These pathways may immunologically activate a recipient cell leading to cellular changes independent of canonical RNAi action. In addition, such stimulation may inhibit the inclusion of exogenous sRNAs into the RNAi pathway by making them targets of antiviral defenses [175]. For example, siRNAs can activate the TLR7 receptor in plasmacytoid dendritic cells [176].

Second, there must be appropriate target mRNA sequences to be regulated via antisense sequence-specific mechanisms. Prediction and validation of intended and actual messenger RNA transcripts that are bound and regulated by a specific sRNA molecule in vivo have been challenging (reviewed in [2]). It is apparent that a combination of the techniques is required to provide convincing evidence of a regulatory relationship. However, groups often rely solely upon one or two of the methods described below, thus leading to inconclusive findings. Bioinformatics methods are most often employed to predict such putative targets. Algorithms exist that take into account the challenges of different kingdom-specific rules for RNAi function [46, 177, 178]. However, in silico approaches are notorious for false positives and missed targets and cannot provide compelling evidence alone [179, 180]. Alternatively, a change in the expression of selected putative targets by a candidate approach can be used after in vivo feeding experiments. However, alone, observed changes in transcript levels do not confirm a direct regulatory interaction. The common practice to demonstrate that a given sRNA directly regulates a target transcript is to use highly engineered reporter constructs that are then exogenously expressed in cell lines with sRNA mimics and inhibitors. To provide more rigorous evidence that an sRNA molecule is both necessary and sufficient to engage a mammalian target mRNA and affect expression, sRNA mimics and inhibitors should be utilized on endogenous targets in whole organisms. An additional approach, not commonly used in this field, uses biochemical methods to identify binding of a given transcript with a given sRNA molecule [181]. Transcriptomics and proteomics would provide a more unbiased approach to discover alterations in post-transcriptional gene expression and should be used when possible. In addition, the use of network biology to find regulatory relationships can provide another unbiased approach for discovery of sRNA-target interactions [182]. Yet, even beyond such bioinformatics, binding experiments, and experiments using heterologous constructs in cell lines, additional experimentation would be required to demonstrate definitively the in vivo function of a putative nucleotide regulatory element [183]. In vivo genetic modification of putative target genes via traditional knock-in techniques or novel ones, such as CRISPR/Cas9, may be required to provide final definitive evidence of a regulatory relationship between a given sRNA and a specific transcript [184].

Third, as previously discussed, functional post-transcriptional gene regulation of mRNA by sRNA requires that a minimum amount of a given sRNA species be taken up by a recipient cell. While dependent on the amount of targeted transcript present, the amount of sRNA required to effect biologically relevant effects on gene expression is currently thought to be 100–10,000 copies per target cell [98–100].

## Technical difficulties and absence of methodological consensus

There is general agreement that sRNAs from dietary sources are observed consistently in mammalian tissues. However, concerns about technical difficulties and a lack of consensus on appropriate methods have led to differences of opinion regarding the robustness, reproducibility, and biologic significance of results [47, 48].

In general, studies to date quantifying dietary sRNAs have relied on an amplification step prior to or associated with measurement. Whether using reverse transcription and quantitative PCR or RNA-sequencing, such data is prone to false positives and bias. Clearly defined limits of detection are critical to excluding the technical “noise” inherent in such assays [185]. The occasional (and possibly non-specific) amplification of a plant sequence at high threshold cycle (Ct) or fractional or single-digit high throughput sequencing (HTS) reads per million of a plant miRNA do not likely represent signal above background.

Variation in the methods used for library preparation, alignment, and analysis can lead to problems of reproducibility in RNA-sequencing, often called “batch effects.” Library preparation methods, particularly biases in amplification [186, 187], can have dramatic impacts on the data and conclusions drawn from them. For example, two recent studies demonstrated that the choice of library preparation kit could influence amounts of a given sRNA detected in matched samples [188, 189]. The output of data from sequence alignment is also heavily dependent on the tools used, with one study finding a threefold difference in miRNAs identified in a given dataset depending on the algorithm used [190].

There is also a disagreement about the correct normalization protocols to use [191]. Many groups favor unrelated sRNA spike-in controls for technical normalization, but endogenous mRNA and sRNA controls for biologic normalization are also important [192], and, although not often employed, a panel of endogenous genes is preferred for this purpose.

A related issue is the reliance on population-based studies, resulting in the amounts of a specific sRNA molecule in an individual cell being mathematically derived instead of empirically determined. Mathematical derivation of a per cell copy number has been very useful in demonstrating the limited feasibility of meaningful uptake of dietary sRNA at the population level as described in previous sections. However, it is conceivable that subpopulations of cells have specialized concentrating mechanisms, which would be obscured at the population level. Yet, only empirical demonstration that levels of a given sRNA are above a 100-copy threshold per cell in a given subpopulation would be able to provide compelling evidence against the current mathematical estimates.

Another key problem is potential contamination, especially associated with ultra-sensitive assays that utilize amplification steps prior to quantification. Widespread contamination has been reported in high throughput sequencing datasets [44] and evidence implicates this issue in the dietary sRNA field since its inception [193]. For example, one group found that sequences corresponding to the monocot sRNA MIR168a were routinely found in datasets [37]. However, the authors pointed out that no realistic biologic rationale existed for the presence of monocot source material in the samples examined. In another example, reexamination of data sets from the Liang et al. study [43] revealed that the sRNA molecule most efficiently taken up was of monocot origin, despite the fact that human subjects in the study had only been fed dicot material. Recently, another group found that over 80% of xenomiRs found in 432 human body fluid sample datasets matched sequences from rodents [65], providing further evidence that the presence of sRNAs from exogenous sources was artifactual rather than diet-derived.

An additional limitation of current approaches is the experimental decoupling of assays that measure the amount of a given sRNA and its activity. sRNA amounts are detected using amplification-dependent methods. Subsequently, the activity of a given sRNA on a given target is demonstrated in a separate system where the sRNA is transfected or expressed at supra-physiologic levels.

A final issue includes the consideration of potential sRNA-independent effects of any diet that could confound interpretation of sRNA activity. Most diets are a complex mixture of macromolecules and micronutrients and ascribing an effect to one component is often quite difficult. For example, Dickinson et al. [26] provided evidence that nutritional intake, not diet-derived sRNAs, were ultimately responsible for the reported alterations in LDL found in the original findings of Zhang et al. [16]. In order to fully address such concerns, comparisons using dietary material from wild-type organisms with mutants engineered to lack a specific sRNA molecule via genetic modification will be required.

A concerted effort to discuss these issues and coalesce around guidelines for future work could bolster any future work in the field of dietary sRNA. Generation of an expert consensus guideline, written by diverse leaders and stakeholders in the field, to define methods to address these technological issues and to provide regulations of experimental design and interpretation for future studies to follow, would be invaluable. Using such a guideline, a consortium and agreement could be established such that any major scientific finding of dietary sRNA uptake discovered by one group would have to be repeated independently by a blinded second group prior to publication. Such an endeavor could dramatically strengthen the reputation and notoriety of the discoveries and elevate the significance of this fledging field, in general. Additionally, such endeavors, which have been quite successful in advancing other fields stalled by controversy [194], could serve as a means to bolster related fields that face similar technological challenges, such as those focused on the physiologic roles of endogenous extracellular sRNAs.

## Conclusions

While the potential impacts of dietary sRNA uptake are exciting, the weight of evidence thus far has demonstrated that generalized dietary sRNA transfer and gene regulation in mammals are neither prevalent nor robust events. While it is possible that more specialized circumstances may allow for such transfer, there is a clear absence of decisive proof. Furthermore, a close examination of current data reported as “supportive” of dietary sRNA uptake typically reveals descriptive phenomenology where multiple interpretations, including technical artifact, could explain the results. Otherwise, a number of follow-up studies have more clearly demonstrated technical artifact and a lack of reproducibility as key confounders. As a result, such issues have substantially and adversely affected general scientific enthusiasm for this field of study.

We propose potential strategies to rectify the absence of consensus on technical issues and our limited mechanistic understanding of the putative steps required for successful modulation of the gene expression by dietary sRNAs. First, the presence of diet-derived sRNAs should be confirmed in tissues and cells of ingesting organisms using methods that do not require amplification for detection. Second, the ability to use sRNAs isolated from the diet directly in assays that measure their RNAi activity should be established. Third, putative sRNA transport, amplification, and concentration systems should be characterized at the molecular level, and rigorous genetic and pharmacologic gain- and loss-of-function experiments should be utilized to demonstrate function. Finally, further progress and enthusiasm in this field will absolutely depend on general and public agreement on methods and controls used in experimental proof as well as blinded and independent replication of any key findings in the future.

## Abbreviations

dsRNA: Double-stranded RNA
GE: Genetically engineered
miRNA: MicroRNA
siRNA: Short-interfering RNA
sRNA: Small RNA
